# Supplementary material for: Multi‐dataset identification of innovative feature genes and molecular mechanisms in keratoconus
Source: J Cell Mol Med. 2024 Sep 19;28(18):e70079. doi: 10.1111/jcmm.70079 (PMC11412914; doi:10.1111/jcmm.70079)
Supplement: Supplementary file 2 — Material S2. [file JCMM-28-e70079-s002.zip › 7.GSEA analysis/1.ARL11/KEGG_LINOLEIC_ACID_METABOLISM.html]

Details for gene set KEGG\_LINOLEIC\_ACID\_METABOLISM[GSEA]

|  || Dataset | ARL11.group.cls#h\_versus\_l |
| Phenotype | group.cls#h\_versus\_l |
| Upregulated in class | l |
| GeneSet | KEGG\_LINOLEIC\_ACID\_METABOLISM |
| Enrichment Score (ES) | -0.40858448 |
| Normalized Enrichment Score (NES) | -1.0923082 |
| Nominal p-value | 0.33530572 |
| FDR q-value | 1.0 |
| FWER p-Value | 0.997 |
Table: GSEA Results Summary

  

Fig 1: Enrichment plot: KEGG\_LINOLEIC\_ACID\_METABOLISM      
 Profile of the Running ES Score & Positions of GeneSet Members on the Rank Ordered List

  

| SYMBOL | TITLE | RANK IN GENE LIST | RANK METRIC SCORE | RUNNING ES | CORE ENRICHMENT || 1 | CYP3A7 | na | 3477 | 0.220 | -0.0339 | No |
| 2 | CYP2C19 | na | 4519 | 0.182 | -0.0091 | No |
| 3 | PLA2G4E | na | 4751 | 0.174 | 0.0366 | No |
| 4 | CYP1A2 | na | 5834 | 0.141 | 0.0480 | No |
| 5 | PLA2G3 | na | 6310 | 0.129 | 0.0731 | No |
| 6 | CYP3A5 | na | 7636 | 0.099 | 0.0647 | No |
| 7 | ALOX15 | na | 8181 | 0.089 | 0.0757 | No |
| 8 | CYP2E1 | na | 8693 | 0.079 | 0.0849 | No |
| 9 | PLA2G12B | na | 9215 | 0.072 | 0.0914 | No |
| 10 | PLA2G2C | na | 13424 | 0.024 | -0.0227 | No |
| 11 | JMJD7-PLA2G4B | na | 14509 | 0.015 | -0.0495 | No |
| 12 | CYP3A4 | na | 14527 | 0.015 | -0.0455 | No |
| 13 | PLA2G4A | na | 17245 | -0.005 | -0.1223 | No |
| 14 | CYP2C9 | na | 17344 | -0.006 | -0.1234 | No |
| 15 | PLA2G6 | na | 17692 | -0.008 | -0.1310 | No |
| 16 | PLA2G12A | na | 17822 | -0.009 | -0.1320 | No |
| 17 | CYP2C8 | na | 18873 | -0.016 | -0.1574 | No |
| 18 | PLA2G2E | na | 19951 | -0.023 | -0.1814 | No |
| 19 | CYP2C18 | na | 20676 | -0.029 | -0.1936 | No |
| 20 | CYP2J2 | na | 22727 | -0.047 | -0.2384 | No |
| 21 | PLA2G10 | na | 23788 | -0.060 | -0.2510 | No |
| 22 | PLA2G2D | na | 27985 | -0.130 | -0.3326 | No |
| 23 | PLA2G2F | na | 30623 | -0.208 | -0.3459 | Yes |
| 24 | PLA2G5 | na | 32093 | -0.264 | -0.3087 | Yes |
| 25 | CYP3A43 | na | 32273 | -0.273 | -0.2315 | Yes |
| 26 | PLA2G2A | na | 32310 | -0.275 | -0.1498 | Yes |
| 27 | PLA2G1B | na | 33526 | -0.362 | -0.0758 | Yes |
| 28 | AKR1B10 | na | 33571 | -0.367 | 0.0333 | Yes |
Table: GSEA details [plain text format]

  

Fig 2: KEGG\_LINOLEIC\_ACID\_METABOLISM      
 Blue-Pink O' Gram in the Space of the Analyzed GeneSet

  

Fig 3: KEGG\_LINOLEIC\_ACID\_METABOLISM: Random ES distribution      
 Gene set null distribution of ES for **KEGG\_LINOLEIC\_ACID\_METABOLISM**
